# Novel *in vivo* Assessment of Unruptured Intracranial Aneurysm Inflammatory Factors

**DOI:** 10.3389/fneur.2020.00439

**Published:** 2020-06-03

**Authors:** Steve M. Cordina, Shant Afarian, William T. Gerthoffer, Anthony Martino, Russell Wilson, Dean K. Naritoku

**Affiliations:** ^1^Department of Neurology, University of South Alabama College of Medicine, Mobile, AL, United States; ^2^Department of Neurosurgery, University of South Alabama College of Medicine, Mobile, AL, United States; ^3^Department of Radiology, University of South Alabama College of Medicine, Mobile, AL, United States; ^4^Department of Biochemistry and Molecular Biology, University of South Alabama College of Medicine, Mobile, AL, United States; ^5^Department of Pharmacology, University of Nevada School of Medicine, Reno, NV, United States; ^6^Department of Pharmacology, University of South Alabama College of Medicine, Mobile, AL, United States

**Keywords:** complement, inflammation, subarachnoid hemorrhage, endovascular, aneurysm

## Abstract

**Background and Purpose:** The growth and eventual rupture of intracranial aneurysms may be due to an underlying inflammatory process as evidenced by pathological examination of aneurysm walls. We hypothesize that unruptured aneurysms have an increased inflammatory milieu within their lumen in comparison to the rest of the cerebral arterial vascular system.

**Methods:** Blood was sampled from unruptured aneurysms in patients presenting for aneurysm coil embolization and C3 and C4 complement values from this serum were compared with complement values in the parent artery.

**Results:** Ten patients were enrolled over 32 months with a mean aneurysm size of 9.1 mm. Compared to control samples drawn from peripheral circulation, there were significant decreases of both C3 (*p* = 0.0003) and C4 (*p* = 0.0063) levels in aneurysmal blood samples.

**Conclusions:** A state of decreased complement indicative of classic pathway activation was found in all tested aneurysms, thus providing evidence of an ongoing process of complement activation in the blood of live, unruptured aneurysm sacs.

## Introduction

Cerebral aneurysm rupture is the primary cause of atraumatic subarachnoid hemorrhages (aSAH). The incidence of reported ruptured aneurysms is about 7.7 in every 100,000 persons per year (about 25,000 individuals per year in the United States), most commonly in people aged 55 years and older. Separately, the incidence of unruptured aneurysms is higher at 15.6 per 100,000 persons per year (1). Aneurysm rupture carries a 40% 30-day risk of mortality (2). The risks of forming aneurysms are associated with family history, genetic background, smoking, trauma, infections, tumors, other coexisting cerebrovascular disorders (for example, arteriovenous malformations) and hypertension. It is known that the possible risk factors for rupture include size, previous history of aSAH, posterior circulation, female sex, advancing age, and smoking. However, given the disparity between the number of ruptured aneurysm incidence and the prevalence of aneurysms, it is felt that other factors could be at play.

Intracranial aneurysms exhibit inflammatory changes that are readily measured during aneurysmal wall pathological examination. The interplay of various factors, including macrophages, T-lymphocytes, IgG, and IgM as well as complement and vascular cell adhesion molecule 1 (VCAM-1) were found in aneurysm walls but not in control vessel walls and is implicated in both extracranial and intracranial aneurysm formation and growth (3); the role of various inflammatory factors was again demonstrated in 2011 when animal models revealed T-cell infiltration, immunoglobulin and complement deposition and activation, and apoptosis within the walls of cerebral aneurysms (4). Baker et al. (5) showed that elevated serum elastase levels were found in patients with both ruptured and unruptured aneurysms with an underlying increased serum elastase-alpha-1 antitrypsin ratio in these patients compared to normal controls. Non-invasive imaging evaluation of aneurysm surface ratios, non-sphericity, and pulsatility indices were also found to be consistent predictive risk factors of rupture up to years before the event in a small patient series (6). A role of smooth muscle cells in the formation, degeneration, and rupture of intracranial aneurysms has also been described (7).

The role of complement in the pathogenesis of aneurysms has also been studied. The weakening of the outer aneurysmal wall in cases of ruptured aneurysms was investigated in two separate studies by Tulamo et al.; the 2006 study (8) revealed the deposition of membrane attack complex (MAC) in the outer walls of ruptured aneurysms, and the 2010 study (9) demonstrated the relative lack of complement-inhibiting factors at the outer aneurysmal wall. A third study (10) revealed that the activation of complement in intracranial aneurysms occurs chronically—rather than acutely—via the classic complement pathway.

It is, therefore, of considerable interest to see whether accurate and reproducible predictions can be made with regard to unruptured aneurysms in order to guide the clinician when an aneurysm should be treated or else watched. We hypothesize that unruptured aneurysms have an inflammatory biochemical milieu. Complement C3 and C4 are established markers of inflammation (11). In this study, we compared aneurysmal vs. parent artery levels of complement to determine if there are measurable differences in complement levels.

## Methods

### Data Sources and Subjects

The study was approved by the IRB at the University of South Alabama (USA IRB 12-274). The University Hospital had, at the time of data collection, an average of 30 ruptured and unruptured aneurysms that presented yearly. Subjects that presented were selected based on aneurysm size and unruptured aneurysm state. Informed consent was obtained for all patients. All aneurysms had to be previously untreated. Aneurysms <10 mm were initially excluded; however, due to limited initial enrollment, the size requirement was changed to more than 5 mm. Demographic and clinical data were obtained for each patient. No primary data or protected health information was transmitted outside the trial database.

### Sample Collection

**Figure 1:**
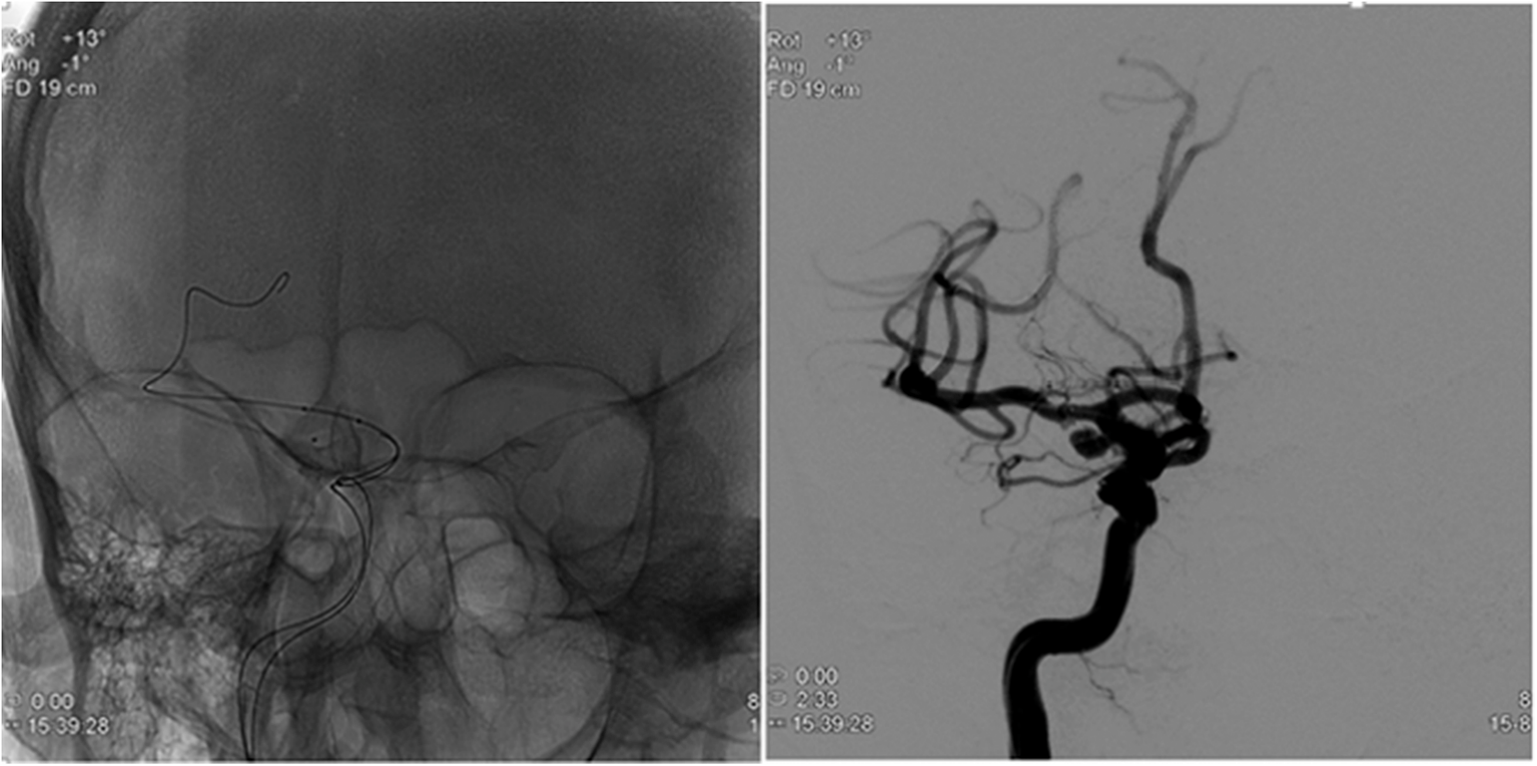
Placement of microcatheter within aneurysm sac for pre-embolization blood sampling.

During the procedure, after induction of anesthesia, right or left common femoral artery access was obtained, through which a guiding catheter (typically Flexor® Shuttle, Cook® Medical, Bloomington, IN, or Neuron™ Max, Penumbra® Inc., Alameda, CA) was navigated to the aneurysm parent artery. Following this, a microcatheter (typically Excelsior® SL-10 ®, Stryker® Neurovascular, Kalamazoo, MI, or Headway® 17 Advanced, MicroVention® Inc., Tustin, CA) was placed within the aneurysm sac in preparation for coil embolization (Figure 1). At this time, a total of 3 mL of blood was drawn from the aneurysm (aneurysm sample) in conjunction with 3 mL blood drawn from the guiding catheter (parent artery sample). The resulting serum was then sent for complement C3 and C4 measurement by quantitative immunoturbidimetry at a reference laboratory (ARUP® Laboratories, Salt Lake City, UT). After this blood draw, the procedure was then completed with aneurysm coil embolization.

### Statistical Analysis

Age in years was expressed as a mean ± standard deviation, and differences between the aneurysm and parent artery control groups were tested for significant difference using the paired *t*-test. Aneurysm size in millimeters was expressed as a mean ± standard deviation. Aneurysm location was described by naming the parent vessel. Differences were considered statistically significant when *P* was ≤ 0.05. All statistical analyses were performed using GraphPad Prism® 6 v. 6.05 (GraphPad Inc., La Jolla, CA).

## Results

### Patient Characteristics

A total of 10 patients were enrolled over a period of 32 months. Nine patients (90%) were female. Seven patients (70%) had a history of recent tobacco smoking, one (10%) had a remote history of smoking, and two patients (20%) were lifelong non-smokers. Six patients (60%) were white, and four patients were black (40%). Nine patients (90%) had a history of hypertension. Patient age ranged from 33 to 71 years, and mean age was 53.2 years (±11.6 years). Five aneurysms (50%) were in the anterior circulation. Aneurysm size ranged from 5 to 14 mm with mean size 9.1 mm (± 2.8 mm) (Table 1).

**Table 1:** Patient demographics and aneurysm characteristics.

**Patient**	**Sex**	**Race**	**Smoking**	**Hypertension**	**Aneurysm size/mm**	**Aneurysm location**
1	M	W	Current	Yes	11	Basilar apex
2	F	W	Current	Yes	10	Right MCA
3	F	W	Current	Yes	10	Right ICA
4	F	B	Current	No	9	Right P-comm
5	F	B	Never	Yes	14	Right ICA
6	F	W	Remote	Yes	7	Basilar apex
7	F	W	Current	Yes	5	Right ICA
8	F	B	Never	No	5	Left ICA
9	F	B	Current	Yes	9	Basilar apex
10	F	W	Current	Yes	11	Basilar apex

*ICA, Internal Carotid Artery; MCA, Middle Cerebral Artery; P-Comm, posterior communicating artery*.

**Figure 2:**
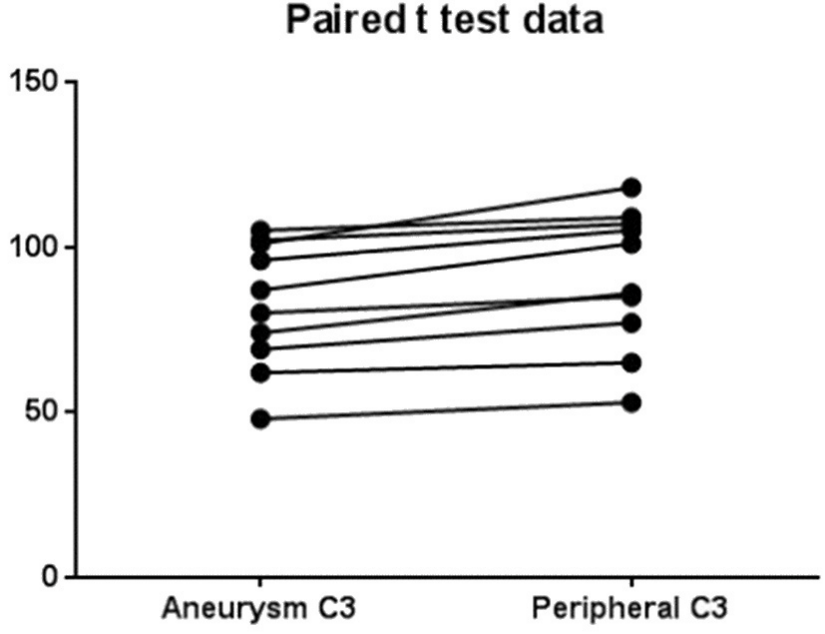
Paired *t*-test data for complement C3 in aneurysm and peripheral blood.

**Figure 3:**
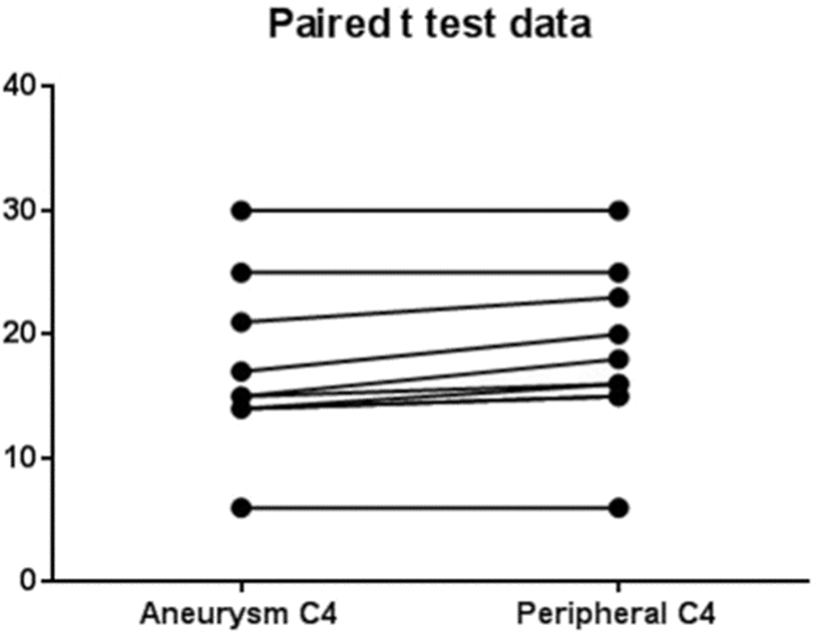
Paired *t*-test data for complement C4 in aneurysm and peripheral blood.

The levels of complement for each subject are shown in Table 2. There was a significant (*p* = 0.0003) decrease in C3 levels in aneurysmal blood samples as compared with intravascular C3 levels. Mean of differences was 8.4, *SD* 4.58, 95% CI 5.13 to 11.7 (Figure 2). There was also a significant (*p* = 0.0063) decrease in C4 levels in aneurysmal blood samples as compared to intravascular C4 levels. Mean of differences was 1.3, *SD* 1.16, 95% CI 0.47 to 2.13 (Figure 3).

**Table 2:** Laboratory values of aneurysm and peripheral C3 and C4.

**Patient**	**Aneurysm C3, mg/dL**	**Peripheral C3, mg/dL**	**Aneurysm C4, mg/dL**	**Peripheral C4, mg/dL**
1	74	86	15	18
2	48	53	6	6
3	96	105	21	23
4	80	85	15	16
5	101	118	14	15
6	69	77	14	16
7	87	101	17	20
8	102	107	30	30
9	103	109	25	25
10	62	65	14	15

## Discussion

The role of inflammation in the pathogenesis of vascular disease has been thoroughly studied. Regarding aneurysms, various inflammatory molecules have been shown to contribute to aneurysm formation and rupture. The complement system is one such system that has been investigated; the preexisting body of work on the complement system in the context of aneurysms has revealed increased complement activity and deposition in cerebral aneurysms. Our work has demonstrated a reduction in intra-aneurysmal levels of C3 and C4 compared to blood samples from control vessels. This reduction can be interpreted as indicative of complement deposition with consequent reduced intra-aneurysmal complement serum levels.

Inflammation causing remodeling of vascular extracellular matrix has been implicated in multiple vascular diseases, including atherosclerosis (12), abdominal aortic aneurysms, intracranial aneurysms (3, 13) as well as arteritis (14). Macrophages, a known source of increased matrix metalloproteinase (MMP) production as well as B- and T-lymphocytes are diffusely present in walls of abdominal aortic aneurysms. Macrophage infiltration has also been shown to be associated with cerebral aneurysm rupture (15, 16). Development of abdominal aortic aneurysms has been linked to MMP release, inflammatory cytokines, and immunoglobulin deposition as well as complement activation in the aneurysm walls. MMPs as well as other proteolytic enzymes have been detected in aneurysm walls and serum of patients with intracranial aneurysms (3). These enzymes lead to a degradation of the vascular wall and disruption of the internal elastic lamina. The presence of histological signs of inflammation both before and after aneurysm rupture certainly supports this. Activated macrophages, T cells, and natural killer cells have been described in areas of aneurysm rupture and severe vessel wall thinning (17).

In this setting, presence of complement C3 and C4 are markers of activation of the classic complement pathway. The studies conducted by Tulamo et al. (8–10) had already implicated the classical complement pathway in the pathogenesis of aneurysms and revealed the role of complement deposition in causing degeneration and rupture of aneurysmal walls. Our findings of decreased intra-aneurysmal complement levels in unruptured aneurysms further support these findings; likewise, these findings support our hypothesis that these decreased levels are due to the deposition of complement on the aneurysm wall.

In addition to complement levels, intra-aneurysmal changes in other inflammatory markers have been shown. In 2013, Chalouhi et al. conducted a study (18) that involved live intra-aneurysmal sampling of blood for the analysis of various inflammatory factors. The study revealed elevated levels of chemokines and chemoattractant cytokines in aneurysmal blood samples.

Taken together, these findings enforce the thought that inflammatory and immune reactions precede intracranial aneurysm rupture. Our study, however, differs in that no previous live intra-aneurysmal sampling for the specific purpose of complement measurement has ever been reported.

The limited number of patients, the testing of only C3 and C4 complement levels, and gender imbalance noted in our series are potential drawbacks in our study. There was a physical limitation of how much blood could be aspirated from the aneurysms (related to time needed to aspirate through the small bore microcatheters and theoretical risk of introducing non-aneurysmal blood in the intrasaccular samples from oversampling). In addition, the core lab required a minimum 3-mL sample for just C3 and C4, leading us to choose only those variables for analysis. Despite these drawbacks, the changes appear robust even with the small number of patients studied. There is a clear decrease in complement levels in aneurysms as compared to their parent arteries regardless of location, age, sex, and race in all the aneurysms sampled and leads us to believe that further enrollment will compound this finding.

## Conclusions, Contribution to the Field

There appears to be evidence of active inflammation even in unruptured aneurysms. This inflammation may be the underlying cause of aneurysm rupture. Treatment of unruptured aneurysms may, in part, require treatment of inflammatory processes. Further study in the relationship of aneurysm formation and rupture in relation to inflammatory processes is warranted.

## Data Availability Statement

All datasets generated for this study are included in the article/Supplementary Material.

## Ethics Statement

The studies involving human participants were reviewed and approved by University of South Alabama. The patients/participants provided their written informed consent to participate in this study.

## Author Contributions

SC, WG, AM, RW, and DN contributed to data collection, sample collection, and manuscript preparation. SA contributed to manuscript editing.

## Conflict of Interest

The authors declare that the research was conducted in the absence of any commercial or financial relationships that could be construed as a potential conflict of interest.

## Supplementary Material

Supplementary Material Datasets.
